# The long-term psycho-social impact of the pandemic on people with intellectual disability and their carers

**DOI:** 10.1177/00207640231174373

**Published:** 2023-05-16

**Authors:** Alexandra Gabrielsson, Meissam Moghaddassian, Indermeet Sawhney, Sophie Shardlow, Samuel Tromans, Paul Bassett, Rohit Shankar

**Affiliations:** 1 Hertfordshire Partnership University NHS Trust, Hatfield, UK; 2 University of Leicester, UK; 3 Leicestershire Partnership NHS Trust, UK; 4 Stats Consultancy, Bucks, UK; 5 Peninsula School of Medicine, University of Plymouth, UK; 6 Cornwall Partnership NHS Foundation Trust, Truro, UK

**Keywords:** COVID-19, developmental disabilities, developmental disorders, mental health, long term harm

## Abstract

**Background:**

People with intellectual disabilities (PWID) are at six times higher risk of death due to COVID-19. To mitigate harm, as a high-risk group, significant social changes were imposed on PWID in the UK. Alongside these changes, the uncertainty of the pandemic influence, caused PWID and their carers to encounter significant stress. The evidence of the pandemic’s psycho-social impact on PWID originates mainly from cross-sectional surveys conducted with professionals and carers. There is little research on the longitudinal psycho-social impact of the pandemic from PWID themselves.

**Aims:**

To examine the long-term psycho-social impact of the pandemic on PWID.

**Methods:**

A cross-sectional survey, following STROBE guidance, of 17 Likert scale statements (12 to PWID and 5 to their carers) to ascertain the pandemic’s psychosocial impact was conducted. Every other PWID open to a specialist Intellectual Disability service serving half a UK County (pop:500,000) was selected. The same survey was re-run with the same cohort a year later. Descriptive statistics, Mann-Whitney, Chi-square and unpaired-t tests were used to compare responses. *S*ignificance is taken at *p* < .05. Comments were analysed using Clarke and Braun’s approach.

**Results:**

Of 250 PWID contacted, 100 (40%) responded in 2020 and 127 (51%) in 2021. 69% (2020) and 58% (2021) reported seeking medical support. Carers, (88%, 2020 and 90%, 2021) noticed emotional changes in PWID they cared for. 13% (2020) and 20% (2021) of PWID had their regular psychotropics increased. 21% (2020) and 24% (2021) had their pro re nata (PRN) medication adjusted. PWID or carers demonstrated no statistically significant variation in responses between themselves from 2020 to 2021. PWID were more likely to report being upset/distressed compared to their carers’ perceptions of them in both years (*p* < .001). Four themes were identified.

**Conclusion:**

This longitudinal study highlights the diverse psycho-social impact of the pandemic on PWID in the UK. The Pandemic’s psycho-social impact has been significantly underestimated.

## Introduction

It is widely accepted in the literature that people with intellectual disability (PWID) experience significant multimorbidity and higher mortality rates when compared to the general population (Glover et al., 2017; LeDer, 2021). The longstanding health disparities and inequity in access to physical health care experienced by PWID compared to the general population have been further amplified in the COVID-19 era (Perera et al., 2020). PWID are more likely to present atypically when infected with COVID-19 and diagnosis is often delayed or missed, and when they do receive hospital care for COVID-19 infection they are far less likely than the general population to be offered interventions such as non-invasive ventilation, intubation or ICU admission (Baksh et al., 2021; Shankar et al., 2023). Such discrepancies in health access for PWID have likely contributed to the COVID-19-related mortality figures reported by NHS England, thought to be as much as six times higher than in the general population (Baksh et al., 2021). Those with moderate to severe intellectual disability (ID) have been found to be particularly vulnerable (Courtenay & Perera, 2020). Moreover, access to routine health services for non-COVID health conditions fell by 20% during the initial phase of the pandemic (Charlesworth et al., 2020). Admission rates for common presentations, including respiratory illness, cancer care and cardiovascular conditions decreased substantially (Shah et al., 2022). The loss of routine health care appointments during the pandemic (Chudasama et al., 2020), raised concerns that complex chronic physical health needs of PWID was being overlooked during the pandemic. The increased risk of infection vulnerability, higher rates of premature mortality, requirement to isolate and the lack of a definitive solution were all a considered source of ongoing psychological concern for PWID and their carers in the first two years of the pandemic.

### Psychological impact of the COVID-19 pandemic on PWID

The rapid spread of the COVID-19 virus and subsequent public health implementations, including nationwide lockdowns, had a detrimental effect on the mental health of the UK population as a whole (Chandola et al., 2022). Given the higher pre-pandemic prevalence of mental illness in PWID compared to the general population (Cooper et al., 2015), many professionals voiced concerns early on of a disproportionately damaging impact on the mental health of PWID of the pandemic (Courtenay & Perera, 2020).

Limited autonomy in daily life and extensive care needs were amongst the concerns highlighted as putting this cohort at higher risk of mental health deterioration, along with cognitive and communicative deficits, which limit understanding and comprehension of changes in social norms and guideline (Courtenay & Perera, 2020).

Recent studies have substantiated the above concerns; The adjustments to daily life, loss of routine, reduced family contact and new public health measures (such as the wearing of face masks and social distancing guidelines), were found to be very distressing for numerous PWID (Frank-Crawford et al.,2021). Gaps in service interventions and challenges associated with supporting the needs of PWID brought on by the pandemic have been hypothesised to underpin the increase in psychotropic prescribing seen for this cohort, resulting in an over-reliance in pharmacological interventions as a compensatory mechanism for gaps in service provision (Naqvi et al., 2021). Such practice puts the person at risk of a whole host of potential side effects, short and long term, which could have detrimental effects on wellbeing and quality of life (Abosi et al., 2018).

### Pandemic impact on carers of PWID

Parents and other carers of PWID have also been disproportionately affected by the pandemic, with literature pointing to a three times higher rate of depression and anxiety in this cohort compared to parents of neurotypical children (Willner et al., 2020). Pressures of adapting to new ways of working and accessing support from health care professionals (i.e. through remote technology) and the need for fast implementation of novel infection control procedures have been found to contribute to high levels of burnout and anxiety amongst those caring for this group of highly vulnerable individuals (Sheehan et al., 2022). Peer-reviewed literature is rich with publications addressing the impact of COVID-19 on the ID population and range from studies exploring professionals’ views (Howkins et al., 2022) the perspective of family and other carers (Tromans et al., 2020), as well as PWID themselves (Rosencrans et al., 2021).

However, there is no study which looks at the psycho-social impact of the pandemic on the same population of PWID and their carers longitudinally. This study is the first to do so in a UK ID service at two points a year apart, during the pandemic.

### Aim

To understand the psycho-social impact of the COVID-19 pandemic on PWID and their carers.

### Methodology

The STROBE Guidance for cross sectional studies was followed (Supplemental Information 1).

#### Survey development

Draft questions for the survey were created collaboratively by three psychiatrists working in a service covering half of a UK county that is, an approximate population of 500,000. It was then passed on to a member of the practice governance team who developed an initial easy-read survey. The draft survey was then shared with the Inclusive Communication team in the County Council, a team dedicated to promoting and supporting delivery of effective communication between PWID and care professionals. They provided guidance on inclusive communication measures and for further adaptations to the survey. The survey (Supplemental Information 2) had two parts comprising of 12 questions intended for the PWID themselves and 5 further questions for their carers. All questions require ‘yes’, ‘no’, or ‘not sure’ responses for ease of communication.

#### Ethics and governance

The project used anonymised pooled data from a single centre. Data was collected as part of ongoing service evaluation and registered as such respectively with the organisation. The NHS Health research authority tool (http://www.hra-decisiontools.org.uk/research/index.html) confirmed no formal NHS Ethics approval was required (Supplemental Information 3). Only the authors who worked for the ID service had access to any patient identifiable information. The collected clinical data was stored anonymously on an EXCEL database and then shared for analysis.

#### Participants and recruitment

The survey was disseminated via post, along with a cover letter and a stamped return envelope. Three NHS consultant psychiatrists’ caseload within an ID service was pooled and listed randomly. Every other patient on the list and their carer was sent the survey in May 2020 and again in May 2021. The same patients and carers were invited to take part on both occasions.

#### Data analysis

##### Analysis of quantitative measures

Baseline demographics across the two years are captured and summarised by mean and standard deviation if normally distributed and continuous variables, with categorical variables summarised by the number and percentage in each category. The analysis focussed on summarising the responses to the survey to all 17 questions and comparing responses between the two years. The same cohort of patients was surveyed in both years. A further analysis was conducted to compare the views of PWID regarding their reported mental state compared to the corresponding views of family/carer respondents by comparing responses to patient Q8 (level of relaxation) and patient Q10 (level of distress) to the equivalent carer questions CQ1(Level of noticed relaxation) and CQ2 (level of noticed stress).

The statistical approach to the response of the cohort at two different intervals was as unpaired subjects. The number and percentage of patients responding in each category in each year is represented. Majority of the questions had three outcome categories: ‘no’, ‘not sure’ and ‘yes’. These questions were assumed to be ordinal outcomes, with the ‘not sure’ response in the middle of the other two categories. Ordinal outcomes were compared between years using the Mann-Whitney test. Questions measured on a binary scale were compared between groups using the Chi-square test. The unpaired t-test was used to compare the age of the two years responses. *S*ignificance of the group differences is taken at *p* < .05.

##### Analysis of qualitative data

Comments were collated and are presented in the Supplemental Material. Analysis of the qualitative data was based on Clarke and Braun’s approach to thematic analysis (Clarke & Braun, 2013) and sought to capture the experiences of carers in supporting PWID through the pandemic. The text was read multiple times to gain an overall familiarity with and understanding of the content. Then, recurring descriptions and comments of interest were highlighted and labelled with a short sentence code which aimed to capture both semantic and latent meaning of the text. This process was revisited multiple times until all relevant data was represented within one of the codes. Following this the codes were collated into named themes and a brief text was added to capture the essence of each theme (Clarke & Braun, 2013).

### Results

#### Quantitative data

Of a total randomly pooled case load of 501, 250 PWID were alternatively selected and survey invites sent. The same 250 were again re-sent the survey the following year. In 2020, 100 survey responses were received that is, 40% response rate. In 2021 there were 127 respondents that is, 51% response rate.

Table 1 summarise the demographics of the PWID who participated in the study, with comparisons between the respondent groups from 2020 and 2021 respectively. Gender was only recorded for the 2021 respondent group, where 56% (*n* = 71*)* of respondents reported being male. The 2020 and 2021 PWID respondent groups did not differ significantly with regards to their distribution of age or level of ID. With regards to level of ID of respondents, the majority were diagnosed with ‘mild ID’ (44%, *n* = 41 in 2020 and 50%, *n* = 61 in 2021).

**Table 1.** Demographic details of the ID patient study populations.

Variable	2020 (*n* =100)		2021 (*n* =127)		*p*-value
	*N*	Summary	*n*	Summary	
Age	99	45.0 ± 13.9	127	46.4 ± 14.6	.48
Gender					
– Female	(No data)	-	127	56 (44%)	-
– Male				71 (56%)	
ID level					
– Mild	94	41 (44%)	121	61 (50%)	.52
– Moderate		38 (40%)		40 (33%)	
– Severe		15 (16%)		20 (17%)	

*Note*. Figures are: mean ± standard deviation, or number (percentage).

Table 2 summarise the service user responses and compares the responses between the year groups. Less than half of patients (44%, *n* = 41 in 2020 and 36%, *n* = 45 in 2021) worried about catching COVID, but patients seemed more concerned about family and friends falling ill (47%, *n* = 44 in 2020 and 46%, *n* = 57 in 2021) and a similar proportion of patients worried who would help care for them if carers fell ill (47% *n* = 26 in 2020 and 40% *n* = 49 in 2021).

**Table 2.** Responses from the study population (PWID) to survey items.

Question	Response	2020 (*n* =100)		2021 (*n* =127)		*p*-value
		*n*	Summary	*n*	Summary	
Q1: Are you worried about getting the Corona Virus?	No	93	39 (42%)	124	59 (48%)	.29
	Not sure		13 (14%)		20 (16%)	
	Yes		41 (44%)		45 (36%)	
Q2: Are you worried about your family, friends or carers getting COVID?	No	94	37 (40%)	124	48 (39%)	.98
	Not sure		13 (14%)		19 (15%)	
	Yes		44 (47%)		57 (46%)	
Q3: Are you worried about who will help you if your carers become unwell?	No	94	38 (40%)	122	43 (35%)	.56
	Not sure		20 (14%)		30 (25%)	
	Yes		36 (47%)		49 (40%)	
Q4: Are you washing your hands more often?	No	96	10 (10%)	122	15 (12%)	.60
	Not sure		7 (7%)		10 (8%)	
	Yes		79 (82%)		97 (80%)	
Q5: Are you keeping your distance from other people?	No	95	22 (23%)	122	19 (16%)	.36
	Not sure		4 (4%)		9 (7%)	
	Yes		69 (73%)		94 (77%)	
Q6: Have you enjoyed being at home more?	No	96	40 (42%)	123	60 (49%)	.35
	Not sure		20 (21%)		22 (18%)	
	Yes		36 (38%)		41 (33%)	
Q7: Have you been doing more activities at home?	No	96	29 (30%)	122	37 (30%)	.94
	Not sure		8 (8%)		9 (7%)	
	Yes		59 (61%)		76 (62%)	
						
Q8: During the lockdown, have you been feeling more relaxed?	No	96	41 (43%)	122	59 (48%)	.34
	Not sure		16 (17%)		21 (17%)	
	Yes		39 (41%)		42 (34%)	
Q9: Is it nice being outside with less people around?	No	96	9 (9%)	122	18 (15%)	.86
	Not sure		19 (20%)		17 (14%)	
	Yes		68 (71%)		87 (71%)	
Q10: Do you feel more upset because of the lockdown?	No	95	21 (22%)	121	30 (25%)	.99
	Not sure		13 (14%)		12 (10%)	
	Yes		61 (64%)		79 (65%)	
Q11: Has changing your daily routine been difficult for you?	No	89	29 (33%)	115	36 (31%)	.96
	Not sure		11 (12%)		16 (14%)	
	Yes		49 (55%)		63 (55%)	
Q12: During lockdown, did you phone your Doctor, 111 or go to Hospital?	No	89	61 (69%)	115	67 (58%)	.13
	Not sure		1 (1%)		1 (1%)	
	Yes		27 (30%)		47 (41%)	

Overall, public health recommendations such as handwashing (82%, *n* = 79) and social distancing (73%, *n* = 69) set out at the beginning of the pandemic were followed, and respondents continued to do so a year on as 80% (*n* = 97) still reported to be washing their hands more and 77% (*n* = 94) made effort to keep distance from others in 2021. Across both years, 71% of respondents (*n* = 68 in 2020 and *n* = 87 in 2021) enjoyed being outside with less people around. Over 60% of respondents (61%, *n* = 59 in 2020 and 62%, *n* = 76 in 2021) engaged in more in-house activities, but fewer reported to enjoy the extra time spent at home (38%, *n* = 36 in 2020 and 33%, *n =* 41 in 2021). Of the respondents, 64% (*n* = 61) felt more upset compared to baseline in 2020 and similar results were returned in 2021 (65%, *n* = 71). Many had to seek support from doctors (69%, *n* = 61 in 2020 and 58%, *n* = 57 in 2021) during the period surveyed.

Table 3 compared the family/carer responses between the two years. Of the family/carer respondents, 88% (*n* = 88) had noticed changes in the emotional wellbeing of the person they cared for in the 2020 survey, and 90% (*n* = 109) in 2021. In 2020, 25% (*n* = 24) of carers had noticed a positive difference with more relaxed clients compared to 20% (n=24) in 2021. Carers reported 46% (*n* = 45) of PWID to be more stressed than usual in 2020 which increased to 52% (*n* = 63) in 2021. In 2020, carers reported that 13% (*n* = 13) of patients had their regular medication increased to help them cope with the pandemic situation, and 20% requiring pharmacological intervention in 2021 (*n* = 25). Pro re nata (PRN) medication was adjusted in a number of cases (21%, *n* = 20 in 2020, 24%, *n* = 26 in 2021). None of the responses to patient and carer questions demonstrated statistically significant variation between themselves for 2020 and 2021.

**Table 3.** Carer responses to survey items.

Question	Response	2020 (*n* = 100)		2021 (*n* = 127)		*p*-value
		*n*	Summary	*n*	Summary	
CQ1: Have you noticed that the service user has been more relaxed/happier than usual?	No	97	61 (63%)	122	85 (70%)	.29
	Not sure		12 (12%)		13 (11%)	
	Yes		24 (25%)		24 (20%)	
CQ2: Have you noticed that the service user has been more stressed than usual?	No	97	45 (46%)	121	45 (37%)	.26
	Not sure		7 (7%)		13 (11%)	
	Yes		45 (46%)		63 (52%)	
CQ3: Has there been an increase in service user’s regular medication to help them cope with the situation?	No	97	81 (84%)	122	92 (75%)	.14
	Not sure		3 (3%)		5 (4%)	
	Yes		13 (13%)		25 (20%)	
CQ4: Has there been any change in the service user’s PRN (as required) medication?	Decrease	95	9 (9%)	117	7 (6%)	.29
	No change		71 (75%)		87 (74%)	
	Increase		15 (16%)		23 (20%)	
CQ5: In the current situation is there anything the learning disability team can do to help the service user?	No	94	74 (79%)	108	82 (76%)	.64
	Yes		20 (21%)		26 (24%)

PWID group were significantly more likely to report themselves as being relaxed compared to their family/carers perceptions of them in 2020 (*p* = .005) and 2021 (*p* < .001). PWID were also significantly more likely to report themselves as being upset/distressed compared to their family/carers’ perceptions of them in both years (*p* < .001).

#### Qualitative data (Table 4)

**Table 4.** Summary of qualitative themes with example comments from respondents.

Themes	Comments 2020	Comments 2021
Theme 1: A need for meaningful pursuits	• Provide more funding for activities and staff levels • More social activities • Offer outside activities that are free	We would like to find somewhere where M can attend for enrichment
Theme 2: Supporting mental health – carers and patient’s	• Having mood swings despite some medication changes • Would like a GP review due to increased anxiety and change to mental health/usual behaviour P has shown more agitation, sudden outbursts at times, due to COVID and lockdown • M’s physical health has not been the best which is also a trigger to increased complex behaviour. Has been closely monitored throughout with psychiatrist • I feel counselling may be a positive option as still struggles with the loss of his nan and grandad • Whilst we appreciate the survey, we think it would have been much better if it wasn’t just about corona virus. This service user has a lot of thinking to deal within his family, which is not COVID-related. I think the survey should have been more general about the service user’s life. • P has shown more agitation, sudden outbursts at times, due to COVID and lockdown.	• Staff require more help to support A’s anxieties, which are also having a knock-on effect on his housemates • M has coped really well during the pandemic. M has done well trying to keep his distance from people. M likes staying in and playing on his gadgets so not going out as much hasn’t really affected M as he prefers staying in. Overall, M has coped really well during this time.
Theme 3: Maintaining link with services	More social calls • A face to face (not telephone only)	Continue with supporting staff members • Continue regular contact with community nurses please
Theme 4: Supporting understanding of the pandemic	• To help understand COVID-19 in more detail at it worries service user	• Does not have the mental capacity to understand what coronavirus actually is
General comments	• Service user is not concerned about COVID 19. Stress levels regarding this have been low	

Of all respondents, 17% (*n* = 17) of carers provided free text responses in 2020 and 9.5% (*n* = 12) in 2021. Four overarching themes were identified, based on the qualitative responses of the study population across the two years and are summarised below.

##### Theme 1: A need for meaningful pursuits

Respondents indicated the need for social activities in daily life to bring enrichment and structure. Funding was mentioned as an obstacle to accessing appropriate activities, with one carer calling for more free activities to be offered.

##### Theme 2: Anxiety-related support for both patients and carers

Carers described the need for both direct and indirect support around managing patient anxieties, which in some cases could affect others around them. However others felt that the patient had coped well during the pandemic and, due to personal preferences for spending time indoors had not been adversely affected by the lockdown restrictions.

##### Theme 3: Maintaining a link with services

Having an established link with and ongoing support (for both patient and carers) from health care services was important amongst the carer respondents. More social calls and opportunities to keep in touch were requested. Carers especially expressed the need to return to face to face review, rather than relying solely on virtual meetings and reviews.

##### Theme 4: Support with understanding the COVID-19 pandemic

Respondents called for further information and education around COVID-19 to be provided by secondary mental health services, to support patient’s understanding of the pandemic.

## Discussion

This study describes the longitudinal experiences of PWID and their carers during the COVID-19 pandemic and draws attention to challenges central to the lockdown experience for this cohort.

The data showed that the majority of respondents had experienced sustained stress and distress during the pandemic, and that levels were higher compared to the pre-pandemic era. Concerns over health (both their own and that of others) and loss of daily activity contributed without significant variation between the two survey points and is consistent with other similar literature (Hatton et al., 2023; Tromans et al., 2020).

Two fifths of PWID worried about who would support them if their carers fell ill. These findings correlate well with other research in the area over the same time period, indicating high levels of stress (Amor et al., 2021; Hatton et al., 2023; McCarron et al., 2021). Amongst measures utilised by PWID to keep themselves safe, more frequent hand washing was most widely adopted at both study points, which is also consistent with findings from other research (Hatton et al., 2023), suggesting that increased hand washing in response to the COVID-19 pandemic was a widely adopted strategy by PWID. Two thirds of respondents to the survey felt more stressed in general than prior to the pandemic and, interestingly, self-reported stress was higher than objective carer reported stress. This is in keeping with other research in the area (McCarron, et al., 2021), which found higher self-reported anxiety levels compared to care-giver assessment of anxiety in PWID. Several factors could contribute to this discrepancy in objective and self-reported levels of wellbeing and stress. Methodological matters related to the composition and accessibility of the survey may have influenced the answers from PWID, due to difficulties in understanding what was being asked from the individual questions. The prevalent emotional state of the PWID on the day of completing the survey could also have influenced their answers, and feelings like anxiety could make it challenging for them to reflect on their longer-term mood state and make comparisons. The level of ID, along with the persons communication skills, are also likely to influence their survey responses, along with potential impairments in episodic memory and temporal sequencing.

In our study, discrepancies are likely to, at least in part, be due to the unpaired nature of the data, but may also highlight challenges in carers objectively assessing emotional states in PWID due to barriers in communication, emotional expression and other cognitive abilities leading to the differential reporting.

Stressors could also be influenced by the care setting (Amor et al., 2021) or spending a lot of time with family could also be a source of conflict and anxiety (Embregts et al., 2022). Two thirds of respondents had engaged with more home-based activities during lockdown, yet only around a third felt they had enjoyed the extra time at home and over half found the abrupt change in their daily routine difficult to cope with. Sudden change to circumstances coupled with limited understanding of the situation can result in behaviours that challenge (Courtenay & Perera, 2020), and put additional strain on the patient-carer relationship and place carers at risk of burnout (Giebel et al., 2022). Family carers might have found change particularly challenging to manage as has been reported by a study which found over half of family carers to be female lone workers, many of whom had their own health problems to manage alongside the needs of the PWID they supported, contributing to care-related anxieties ((Taggart et al., 2012).

The qualitative data obtained was in keeping with the above with the emphasis being on management of anxiety and support with activities. In addition, feedback also suggests that expectations on services may have shifted somewhat during the pandemic, with higher emphasis on providing information, reassurance and prevention of social exclusion. Lake et al. (2021) reported that PWID found themselves flooded by information and misinformation from all directions during the pandemic. This resulted in feelings of anxiety and of being overwhelmed. This could perhaps in part explain the shift in expectation onto secondary mental health services towards providing clear, reliable easy read information and guidance. Our findings on the importance of maintaining links with and support from mental health services were consistent with their findings (Lake et al., 2021).

### Strengths and limitations

A strength of this study is that PWID were asked directly about their views on a range of COVID-19-related issues, rather than family/carers alone being asked. Furthermore, the PWID element of the survey was developed in an easy-read format, with input from an inclusive communication team to ensure that the material was appropriate for the study population. This could explain the high return rates (40% in 2020 and 51% in 2021). This survey included only PWID who are currently engaged with health and or social care services and there is a lack of input from other more marginalised groups of PWID such as those who are supported in inpatient mental health and forensic settings, those who are disengaged with health support and those who are homeless. This makes it difficult to draw generalised conclusions from our results, which we accept as a limitation of the study.

There are numerous other limitations to this study. Certain level of assistance/contribution from carers to PWID in completing the patient-directed questions for those with more severe forms of ID cannot be ruled out. Collection of baseline demographic data was minimal missing issues such as ethnicity, co-morbid illness and current medication etc. It was not possible to pair responses from the two years surveyed as the plan for a follow up survey was done post the first survey. It was decided that the 2^nd^ survey should be similar to the first irrespective of weakness identified post completion of the first survey. Type of care setting was not specified, which limits the conclusions that can be drawn from the data. Closed questions have been utilised in the survey and might have introduced bias into responses but were thought the most effective to elucidate response from the target population. Family/carer qualitative responses tended to have more of a direct health-related focus, rather than being more centred around social elements, such as day service provision. This may be attributable to them having received the survey from representatives of their healthcare trust, rather than social services for example, or it may simply reflect their priority concerns at the time period of the surveys. Additionally, with respect to the changes in medication regime reported in this study, we do not have an equivalent time period outside of the COVID-19 pandemic to act as a comparator. Finally, this was a regional study and generalisability of its results is not definite.

## Conclusion

PWID and their carers report holding broadly similar views on COVID-19 over time on a variety of psycho-social issues, including COVID-related worry, home-based activities, compliance with social distancing, levels of upset, levels of relaxation, the impact of a change in their routine, and their healthcare-seeking behaviours. A similar trend is observed for family/carers with respect to their responses relating to the PWID they care for on issues including worry, relaxation levels, and medication use. However, family/carers appear to struggle to understand the emotional states of the PWID they care for, and this is an area of potential interest for future research.

## Supplemental Material

sj-docx-1-isp-10.1177_00207640231174373 – Supplemental material for The long-term psycho-social impact of the pandemic on people with intellectual disability and their carers Click here for additional data file. Supplemental material, sj-docx-1-isp-10.1177_00207640231174373 for The long-term psycho-social impact of the pandemic on people with intellectual disability and their carers by Alexandra Gabrielsson, Meissam Moghaddassian, Indermeet Sawhney, Sophie Shardlow, Samuel Tromans, Paul Bassett and Rohit Shankar in International Journal of Social Psychiatry

sj-docx-2-isp-10.1177_00207640231174373 – Supplemental material for The long-term psycho-social impact of the pandemic on people with intellectual disability and their carers Click here for additional data file. Supplemental material, sj-docx-2-isp-10.1177_00207640231174373 for The long-term psycho-social impact of the pandemic on people with intellectual disability and their carers by Alexandra Gabrielsson, Meissam Moghaddassian, Indermeet Sawhney, Sophie Shardlow, Samuel Tromans, Paul Bassett and Rohit Shankar in International Journal of Social Psychiatry

sj-docx-3-isp-10.1177_00207640231174373 – Supplemental material for The long-term psycho-social impact of the pandemic on people with intellectual disability and their carers Click here for additional data file. Supplemental material, sj-docx-3-isp-10.1177_00207640231174373 for The long-term psycho-social impact of the pandemic on people with intellectual disability and their carers by Alexandra Gabrielsson, Meissam Moghaddassian, Indermeet Sawhney, Sophie Shardlow, Samuel Tromans, Paul Bassett and Rohit Shankar in International Journal of Social Psychiatry

## References

[bibr1-00207640231174373] Abosi O. Lopes S. Schmitz S. Fiedorowicz J. G. (2018). Cardiometabolic effects of psychotropic medications. Hormone Molecular Biology and Clinical Investigation, 36(1), 10.1515/hmbci-2017-0065/j/hmbci.2018.36.issue-1/hmbci-2017-0065/hmbci-2017-0065.xml; https://pubmed.ncbi.nlm.nih.gov/29320364/PMC6818518 29320364

[bibr2-00207640231174373] Amor A. M. Navas P. Verdugo M. Á. Crespo M. (2021). Perceptions of people with intellectual and developmental disabilities about COVID-19 in Spain: A cross-sectional study. Journal of Intellectual Disabilities, 65, 381–396. 10.1111/jir.12821 PMC8013361 33555099

[bibr3-00207640231174373] Baksh R. A. Pape S. E. Smith J. Strydom A. , (2021). Understanding inequalities in COVID-19 outcomes following hospital admission for people with intellectual disability compared to the general population: A matched cohort study in the UK. BMJ Open, 11(10), e052482. 10.1136/bmjopen-2021-052482 PMC8491000 34607870

[bibr4-00207640231174373] Chandola T. Kumari M. Booker C. L. Benzeval M. (2022). The mental health impact of COVID-19 and lockdown-related stressors among adults in the UK. Psychological Medicine, 52, 2997–3006. 10.1017/S0033291720005048 33280639 PMC7783135

[bibr5-00207640231174373] Charlesworth A. Watt T. Thorlby R. (2020). Early insight into the impacts of COVID-19 on care for people with long-term conditions. The Health Foundation.

[bibr6-00207640231174373] Chudasama Y. V. Gillies C. L. Zaccardi F. Coles B. Davies M. J. Seidu S. Khunti K. (2020). Impact of COVID-19 on routine care for chronic diseases: A global survey of views from healthcare professionals. Diabetes & Metabolic Syndrome, 14(5), 965–967. 10.1016/j.dsx.2020.06.042 32604016 PMC7308780

[bibr7-00207640231174373] Clarke V. Braun V. (2013). Teaching thematic analysis: Overcoming challenges and developing strategies for effective learning. The Psychologist, 26(2), 120–123.

[bibr8-00207640231174373] Cooper S. A. McLean G. Guthrie B. McConnachie A. Mercer S. Sullivan F. Morrison J. (2015). Multiple physical and mental health comorbidity in adults with intellectual disabilities: Population-based cross-sectional analysis. BMC Family Practice, 16, 110. 10.1186/s12875-015-0329-3 26310664 PMC4551707

[bibr9-00207640231174373] Courtenay K. Perera B. (2020). COVID-19 and people with intellectual disability: Impacts of a pandemic. Irish Journal of Psychological Medicine, 37(3), 231–236. 10.1017/ipm.2020.45 32404232 PMC7287305

[bibr10-00207640231174373] Embregts P. J. C. M. van Den Bogaard K. J. H. M. Frielink N. Voermans M. A. C. Thalen M. Jahoda A. (2022). A thematic analysis into the experiences of people with a mild intellectual disability during the COVID-19 lockdown period. International Journal of Developmental Disabilities, 68(4), 578–582. 10.1080/20473869.2020.1827214 35937180 PMC9351556

[bibr11-00207640231174373] Frank-Crawford M. A. Hallgren M. M. McKenzie A. Gregory M. K. Wright M. E. Wachtel L. E. (2021). Mask compliance training for individuals with intellectual and developmental disabilities. Behavior Analysis in Practice, 14(4), 883–892. 10.1007/s40617-021-00583-7 34150186 PMC8204602

[bibr12-00207640231174373] Giebel C. Hanna K. Marlow P. Cannon J. Tetlow H. Shenton J. Faulkner T. Rajagopal M. Mason S. Gabbay M. (2022). Guilt, tears and burnout-Impact of UK care home restrictions on the mental well-being of staff, families and residents. Journal of Advanced Nursing, 78(7), 2191–2202. 10.1111/jan.15181 35188292 PMC9303866

[bibr13-00207640231174373] Glover G. Williams R. Heslop P. Oyinlola J. Grey J. (2017). Mortality in people with intellectual disabilities in England. Journal of Intellectual Disability Research, 61(1), 62–74. 10.1111/jir.12314 27489075

[bibr14-00207640231174373] Hatton C. Caton S. Bradshaw J. Gillooly A. Jahoda A. Kelly R. Maguire R. Oloidi E. Taggart L. Todd S. Hastings R. (2023). Coronavirus and people with learning disabilities study wave 4 results: February 2023 (Full report). University of Warwick.

[bibr15-00207640231174373] Howkins J. Hassiotis A. Bradley E. Levitas A. Sappok T. Sinai A. Thakur A. Shankar R. (2022). International clinician perspectives on pandemic-associated stress in supporting people with intellectual and developmental disabilities. BJPsych open, 8(3), e84. 10.1192/bjo.2022.49 PMC9059730 35431024

[bibr16-00207640231174373] Lake J. Jachyra P. Volpe T. Lunsky Y. Magnacca C. Marcinkiewicz A. Hamdani Y. (2021). The wellbeing and mental health care experiences of adults with intellectual and developmental disabilities during COVID-19. Journal of Mental Health Research in Intellectual Disabilities, 14(3), 285–300. 10.1080/19315864.2021.1892890

[bibr17-00207640231174373] McCarron M. McCausland D. Luus R. Allen A. Sheerin F. Burke E. McGlinchy E. Flannery F. McCallion P. (2021). The impact of coronavirus disease 2019 (COVID-19) on older adults with an intellectual disability during the first wave of the pandemic in Ireland. HRB Open Research, 4, 93. 10.12688/hrbopenres.13238.2 35112049 PMC8772523

[bibr18-00207640231174373] Naqvi D. Perera B. Mitchell S. Sheehan R. Shankar R. (2021). COVID-19 pandemic impact on psychotropic prescribing for adults with intellectual disability: An observational study in English specialist community services. BJPsych open, 8(1), E7. 10.1192/bjo.2021.1064 PMC8649353 34865678

[bibr19-00207640231174373] Perera B. Laugharne R. Henley W. Zabel A. Lamb K. Branford D. Courtanay K. Alexander R. Purandare K. Wijeratne A. Radhakrishnan V. McNamara E. Daureeawoo Y. Sawhney I. Scheepers M. Taylor G. Shankar R. (2020). COVID-19 deaths in people with intellectual disability in the UK and Ireland: descriptive study. BJPsych open, 6(6), e123. 10.1192/bjo.2020.102 PMC7562776 33059790

[bibr20-00207640231174373] Rosencrans M. Arango P. Sabat C. Buck A. Brown C. Tenorio M. Witwer A. (2021). The impact of the COVID-19 pandemic on the health, wellbeing, and access to services of people with intellectual and developmental disabilities. Research in Developmental Disabilities, 114, 103985. 10.1016/j.ridd.2021.103985 34049229 PMC9758885

[bibr21-00207640231174373] Shah S. A. Brophy S. Kennedy J. Fisher L. Walker A. Mackenna B. Curtis H. Inglesby P. Davy S. Bacon S. Goldacre B. Agrawal U. Moore E. Simpson C. R. Macleod J. Cooksey R. Sheikh A. Katikireddi S. V. , (2022). Impact of first UK COVID-19 lockdown on hospital admissions: Interrupted time series study of 32 million people. EClinicalMedicine, 49, 101462. 10.1016/j.eclinm.2022.101462 PMC9121886 35611160

[bibr22-00207640231174373] Shankar R. Perera B. Roy A. Courtenay K. Laugharne R. Sivan M. (2023). Post-COVID syndrome and adults with intellectual disability: Another vulnerable population forgotten? The British Journal of Psychiatry, 222(1), 1–3. 10.1192/bjp.2022.89 35766219

[bibr23-00207640231174373] Sheehan R. Dalton-Locke C. Ali A. Vera San Juan N. Totsika V. Hassiotis A. (2022). Effects of the COVID-19 pandemic on mental healthcare and services: Results of a UK survey of front-line staff working with people with intellectual disability and/or autism. BJPsych Bulletin, 46(4), 201–207. 10.1192/bjb.2021.52 PMC9768507 33977886

[bibr24-00207640231174373] Taggart L. Truesdale-Kennedy M. Ryan A. McConkey R. (2012). Examining the support needs of ageing family carers in developing future plans for a relative with an intellectual disability. Journal of Intellectual Disabilities, 16, 217–234. 22890999 10.1177/1744629512456465

[bibr25-00207640231174373] LeDer (2021). The Learning from lives and deaths—People with a learning disability and autistic people (LeDeR) Report. Retrieved October 23, 2022, from https://www.kcl.ac.uk/news/2021-leder-report-into-the-avoidable-deaths-of-people-with-learning-disabilities

[bibr26-00207640231174373] Tromans S. Kinney M. Chester V. Alexander R. Roy A. Sander J. W. Dudson H. Shankar R. (2020). Priority concerns for people with intellectual and developmental disabilities during the COVID-19 pandemic. BJPsych open, 6(6), e128. 10.1192/bjo.2020.122 PMC7609203 33118913

[bibr27-00207640231174373] Willner P. Rose J. Stenfert Kroese B. Murphy G. H. Langdon P. E. Clifford C. Hutchings H. Watkins A. Hiles S. Cooper V. (2020). Effect of the COVID-19 pandemic on the mental health of carers of people with intellectual disabilities. Journal of Applied Research in Intellectual Disabilities, 33(6), 1523–1533. 10.1111/jar.12811 32885897

