# Supplementary figures and images for: The long-term psycho-social impact of the pandemic on people with intellectual disability and their carers
Source: Int J Soc Psychiatry. 2023 May 16;69(7):1781–9. doi: 10.1177/00207640231174373 (PMC10191827; doi:10.1177/00207640231174373)

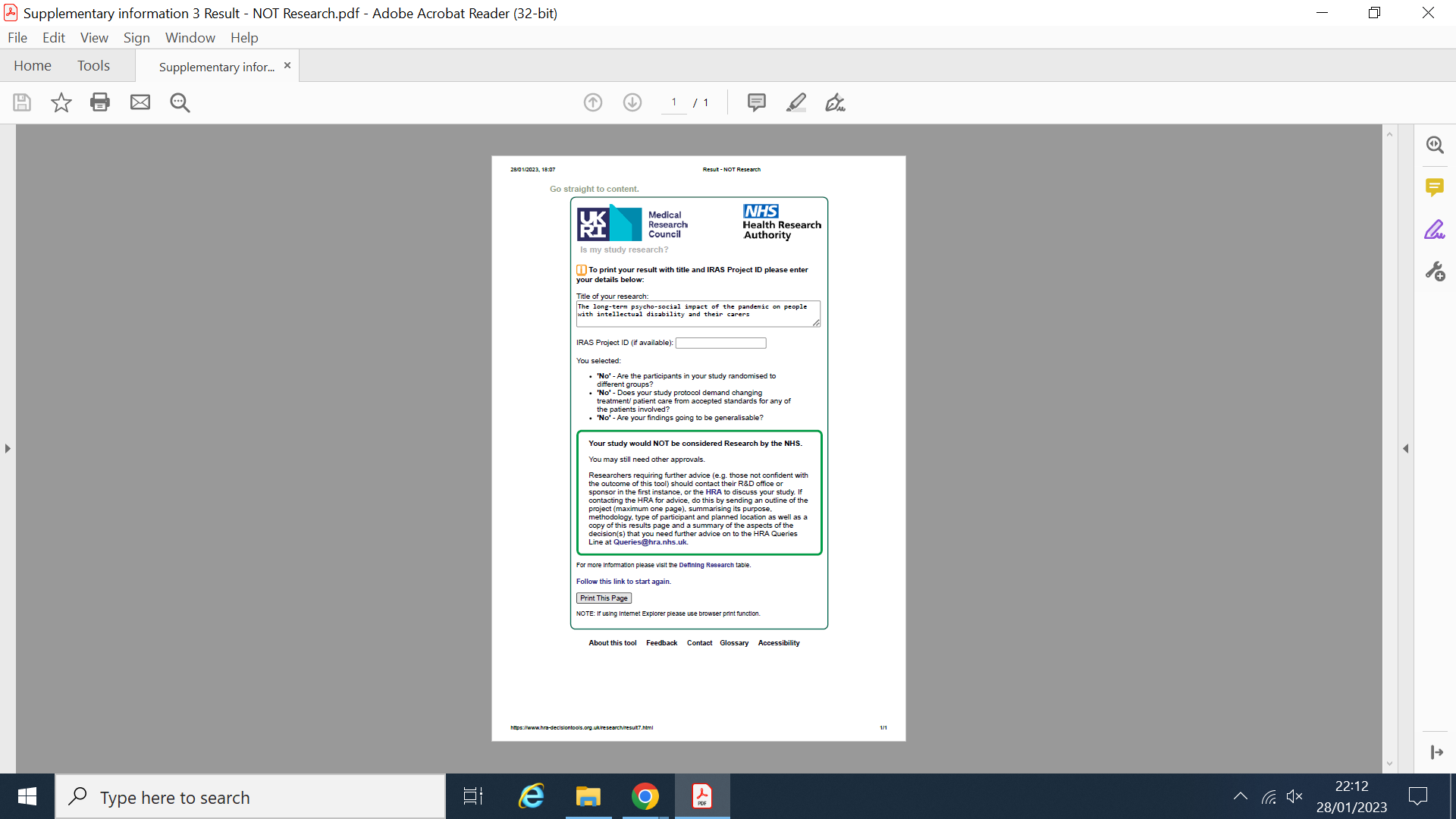

Supplement: sj-docx-3-isp-10.1177_00207640231174373 – Supplemental material for The long-term psycho-social impact of the pandemic on people with intellectual disability and their carers [file sj-docx-3-isp-10.1177_00207640231174373.docx]
